# A qualitative study of sexuality and contraceptive choice among women using withdrawal

**DOI:** 10.1038/s41443-025-01148-w

**Published:** 2025-08-02

**Authors:** Gabrijela Simetinger

**Affiliations:** 1 Department of Gynaecology and Obstetrics, General Hospital Novo mesto, Novo mesto, Slovenia; 2 Faculty of Medicine, University of Ljubljana, Ljubljana, Slovenia

**Keywords:** Quality of life, Health care

## Abstract

This study examines the impact of withdrawal on women’s sexuality and its continued use in Slovenia despite modern contraceptive alternatives. Through in-depth interviews with 45 Slovenian women using withdrawal, findings reveal significant effects on sexual satisfaction, particularly pleasure and orgasm. Many participants reported decreased sexual desire due to pregnancy fears, while concerns about their partner’s performance affected arousal. Difficulties in achieving orgasm were common, with some experiencing reduced intensity and frequency. The decision to use withdrawal was often shaped by male preference, limited access to other contraceptive methods, and societal judgment. Some women preferred withdrawal for its perceived naturalness, aligning with a cultural shift toward naturalism, which may reinforce traditional gender roles and sexual norms. Historically, withdrawal was a widely accepted contraceptive method influenced by social norms and a lack of alternatives. The contemporary preference for natural contraception continues despite its risks, demonstrating how cultural beliefs shape reproductive choices. The findings underscore the need for comprehensive contraceptive counselling that acknowledges both personal and societal factors affecting women’s decisions. Addressing these influences can help ensure informed choices that enhance both sexual well-being and reproductive autonomy.

## Introduction

Withdrawal, or ‘*coitus interruptus’*, remains a widely used traditional contraceptive method globally, with 53 million users representing 5.5% of all contraceptive users in 2020 [1]. Despite advances in and accessibility to modern contraceptives, withdrawal usage persists, with 9% of European couples and a significant portion of Eastern European populations continuing to employ this method [1]. In Slovenia, we have many modern contraceptive methods (contraceptive pills, injections, vaginal contraceptive rings, patches and intrauterine systems and devices) available, and they are all free of charge if a person has basic health insurance. Still, in 2022, among 2996 women who had an abortion, 2097 reported not using any contraception, and 257, or 45% of the remainder, used withdrawal contraception [2]. Similarly, withdrawal-related induced abortions constitute the highest percentage in Italy (39%) [3] and substantial proportions among immigrants in Sweden (25%) [4].

The preference for withdrawal over hormonal contraceptives is increasing among Slovenian women [5], which reflects a broader European trend favouring natural contraceptive methods. This shift warrants a deeper investigation into the reasons for choosing withdrawal, which does not protect against sexually transmitted infections and particularly given its less effective nature; the World Health Organization cites a 4% failure rate with perfect use and 22% with typical use [6].

Withdrawal’s advantages include no cost, no chemical involvement, and no interference with lactation, appealing to those needing immediate or temporary contraception, particularly among populations with religious or philosophical reservations about other methods [7]. However, withdrawal places significant responsibility on male partners, requiring high self-control and often shifting focus from pleasure to performance, with a potential impact on sexual satisfaction [8, 9].

A systematic review and meta-analysis focusing on postpartum contraceptive use among women in low- and middle-income countries identified that withdrawal is frequently adopted due to perceived low pregnancy risks associated with breastfeeding and postpartum amenorrhea. These perceptions are often coupled with concerns regarding the side effects of alternative contraceptive methods and the lack of adequate family planning counselling [10]. Concurrently, a multicentre qualitative study in the United States revealed that women under the age of 25 favour withdrawal, citing its non-invasive nature and perceived lack of harmful effects on the body [11]. Similarly, an Australian study [12] indicated that young women regard withdrawal and similar methods as preferable to hormonal and more invasive contraceptive strategies. While generally satisfied, some participants noted that their reliance on less effective methods stemmed from a scarcity of alternatives, negative experiences with hormonal contraceptives, insufficient information about other options, and challenges in accessing diverse methods [12]. Additionally, qualitative in-depth interviews with 38 Black and Latino women aged 18–24 highlighted that many opt for withdrawal to enhance intimacy with their partners and because they perceive condoms as redundant in monogamous relationships or wish to accommodate their partners’ preferences for increased sexual pleasure [13].

Although numerous studies have examined factors influencing contraceptive choices, the specific impact of withdrawal on women’s sexuality remains underexplored [8, 14, 15]. Existing research has not sufficiently addressed why withdrawal persists despite the availability of modern contraceptive methods or how societal trends promoting naturalistic lifestyles influence its continued use. This gap limits a comprehensive understanding of the implications of withdrawal for women’s sexual well-being and reproductive health. This study aims to analyse the impact of withdrawal on women’s sexual activity and satisfaction, as well as the underlying reasons for its continued use among Slovenian women. By examining these factors within a socio-cultural context, this research contributes to a more nuanced understanding of withdrawal’s role in reproductive health. It informs the development of more effective contraceptive counselling and public health strategies.

## Subjects and methods

A qualitative study was conducted using in-depth, semi-structured interviews. All participants used withdrawal at some point in their lives. The questionnaire was pre-tested through a pilot study. Ethics approval (FZV-81/2015) was received from the Research Ethics Committee of the University of Novo mesto.

### Procedures

This qualitative study employed purposive sampling to recruit participants through Slovenian gynaecologists, who identified women based on predefined inclusion criteria: geographic location, age, sexual orientation (heterosexual, bisexual, or other), and willingness to participate. Eligible participants were women attending preventive gynaecological examinations (e.g., cervical cancer screening or contraceptive consultations) who had used withdrawal at some point in their lives. Of the 45 women approached, none declined participation; however, those who had never used withdrawal as a contraceptive method were excluded. Data collection was conducted in two phases: before the COVID-19 pandemic in 2020 and again in 2024.

Thematic areas for the structured thematic interviews were identified based on a review of relevant scientific literature and the study’s aims. We focused on key topics frequently addressed in prior research on the influence of withdrawal on sexuality, reasons for use, as well as on themes aligned with our specific research questions. As a result, the interviews were structured around the following thematic areas: (1) Sexuality and withdrawal, examining the impact of withdrawal on sexual satisfaction, physiological and emotional changes, and comparative experiences with modern contraceptive methods; and (2) Motivations for use, exploring participants’ reasons for choosing withdrawal, their perceptions of its effectiveness, and the factors influencing continued use despite the availability of alternative methods, including the role of gynaecological consultations. These themes provided a framework for both the interview questions and the subsequent qualitative analysis.

Informed consent was obtained before data collection. Interviews were conducted individually in a private setting and audio-recorded, ensuring confidentiality. Participants chose pseudonyms to maintain anonymity. Audio recordings were transcribed verbatim, anonymised, and assigned unique identifiers to eliminate personally identifiable information. All interviews were conducted by the first author, a researcher with formal training and extensive experience in qualitative data collection, who had no prior personal or professional relationship with any participant.

Data collection concluded upon reaching theoretical saturation, the point at which no new themes or insights emerged, and responses became repetitive. This approach aligns with established principles of qualitative research, ensuring the comprehensiveness and validity of the findings.

### Sample

The sample consisted of 45 women, aged 20–65, with an average age of 39.5 (SD 10.1). Of these participants, 44 described themselves as heterosexual, and one as bisexual. The educational backgrounds of the women varied: 25 participants (55.6%) had completed high school, 12 (26.7%) had attained university education, four had completed higher or higher vocational school, and four had a postgraduate degree (Table 1).

**Table 1:** Sociodemographic characteristics of the sample (*N* = 45).

Characteristic	n (%)
Education	
Completed high school	25 (55.6)
Completed higher school	4 (8.9)
University education	12 (26.7)
Postgraduate degree	4 (8.9)
**Age, mean (SD)**	39.5 (10.1)
Employment status	
Higher-education student	6 (13.3)
Employed	35 (77.8)
Unemployed	3 (6.7)
Other (freelance)	1 (2.2)
Relationship status	
Married	22 (48.9)
Extramarital	14 (31.1)
Single	9 (20.0)
Currently in a relationship	
Yes, more than one year	40 (88.9)
Yes, up to one year	2 (4.4)
No	3 (6.7)
Sexual orientation	
Heterosexual	44 (97.8)
Bisexual	1 (2.2)
Other	0 (0.0)
Place of residence	
City	23 (51.1)
Countryside	22 (48.9)

### Data analysis

Thematic analysis was conducted using Braun and Clarke’s six-phase framework to ensure a systematic and rigorous examination of the qualitative data [16]. The process included familiarisation, coding, theme development, reviewing, defining, and reporting. Themes were iteratively refined through cross-checking against the dataset to ensure coherence and representativeness. Multiple researchers independently coded the data to enhance reliability and validity, resolving discrepancies through a consensus-based approach. Data triangulation was employed by integrating multiple perspectives and cross-verifying participant responses.

## Results

Of the 45 women using withdrawal, 28 (62.2%) relied on it as their primary contraceptive method at some point. Twenty-three (73.3%) then used it incidentally due to a lack of alternatives, while one (2.2%) combined it with more reliable methods. Partner influence was significant, with 14 (31.1%) women learning about withdrawal from their partners and eight (17.8%) having it chosen solely by their partner. Nine (20%) participants experienced unintended pregnancies while using withdrawal; three (6.7%) resulted in childbirth, while six (13.3%) were terminated.

### Influence of withdrawal on sexuality

Participants’ experiences with withdrawal varied in terms of sexual desire, arousal, and orgasm (Table 2). Eighteen (40%) participants reported no impact on sexual desire, while 14 (31.1%) experienced a decrease due to fear of getting pregnant. Twenty-three (51.1%) of the participants believed that sexual intercourse was more satisfying when using modern contraceptive methods. Two (4.4%) reported increased desire, attributing it to sexual dissatisfaction and the need to prolong intercourse.

**Table 2:** Thematic analysis with categories, codes and quotes on the perception of sexuality in women using withdrawal.

Themes	Categories	Codes	Quotes from participants
Influence of withdrawal on sexuality	Sexual desire	Positive - higher	*As I am not fully satisfied, I could go on and on*. (Interviewee 14)
		Neutral - the same	*It doesn’t have any impact*. (Interviewee 15)
		Negative - lower	*It was different. I had no desire*. (Interviewee 16)
	Arousal	Reluctance of the partner’s withdrawal	*You don’t want to, but at a crucial moment, a brake kicks in*. (Interviewee 17)
		Occasional reluctance of the partner’s withdrawal	*But when you have the greatest desire, and there are risky days, you have to be careful*. (Interviewee 18)
		Indifference	*Once I decided that we were going to do that, I totally gave in*. (Interviewee 19)
		Low arousal – a wish for intercourse to be over quickly for fear of getting pregnant	*I was just waiting for him to do get it over with*. (Interviewee 3)
	Pleasure	Neutral – no impact	*He knows how to stimulate me in a different way because he is like that, so he gets involved with me afterwards*. (Interviewee 20)
		Negative affect	*First there is a lack of relaxation… the pleasure is not the same, it’s lower, and the climax is smaller, or how should I say it, there is less pleasure*. (Interviewee 21)
	Orgasm	Neutral	*I wouldn’t say so, because usually, I’ve had an orgasm before that*. (Interviewee 20)
		Negative affect	*There was no orgasm; there was an interruption, and then I couldn’t relax anymore*. (Interviewee 22)
		Fake orgasm	*I faked it*. (Interviewee 23)
		Orgasms frequency	*I can have an orgasm, but if the intercourse goes on without interruption, I can have more of them*. (Interviewee 17)
		Orgasm intensity	*There were fewer orgasms, and they were less intense*. (Interviewee 24)
	Sexual repertoire	Sex play	*Not for this purpose, not to prevent pregnancy, but for fun*. (Interviewee 25)
Reasons for use	Forgotten contraception		*If you forget to take the pill*. (Interviewee 26)
	Hormones, medically contraindicated		*I had an inflammation when I was using the coil. Due to liver infection with hepatitis, I was not allowed to use the pills*. (Interviewee 27)
	Tradition	Adequate contraception	*When you’re young, you think that’s enough*. (Interviewee 28)
		Widespread use	*A few of my colleagues use it to prevent getting pregnant*. (Interviewee 29)
		Gynaecologist’s advice	*My gynaecologist said: “Your husband should be careful.”* (Interviewee 30)
		Occasional use	*There was no other option at that moment*. (Interviewee 31)
	Glorifying the natural	The concept of the natural	*I am more of this view that everything should be natural*. (Interviewee 32)
		Harms of hormonal contraception: ‐ Aversion ‐ Aversion during pregnancy	*I decided to have withdrawal, and I got pregnant. In a way I felt that “contraception could harm me” in my future pregnancy*. (Interviewee 33)
	Encourages switching of partners		*Because the pills were taken by the girls who had more boyfriends*. (Interviewee 34)

Regarding arousal, 19 (42.2%) participants expressed reluctance for their partners to withdraw, whereas eight (17.8%) experienced strong aversion due to extreme fear of getting pregnant. In terms of sexual pleasure, 14 (31.1%) women reported no impact, while 11 (24.4%) noted reduced pleasure.

Fifteen (33.3%) participants reported no change in orgasm when using withdrawal—however, most experienced difficulties, including delayed climax, reduced intensity, or inability to orgasm. Multi-orgasmic 2 (4.4%) participants noted a decrease in orgasm frequency. One (2.2%) participant used withdrawal in combination with other methods to enhance sexual pleasure.

### Reasons for using withdrawal

The primary reasons women choose to withdraw are related to tradition (Table 2). Thirteen (28.9%) participants saw it as a standard contraceptive method, and three (6.7%), were influenced by their peers; another three (6.7%) followed gynaecological advice, and three (6.7%) others resorted to withdrawal for lack of other options.

A few 4 (8.9%) participants highlighted apprehensions regarding the potential impacts of hormonal contraceptives on future pregnancies. Additional reasons were practical oversight (missed a birth control pill or forgot condoms), medical contraindications (liver damage, pelvic inflammatory disease) and sterilisation restriction for women under 35 according to Slovenian regulations (Table 2).

## Discussion

The study was designed to fill research gaps regarding the effects on women’s sexuality and the reasons for their continued use despite the availability of modern contraceptives in the context of Slovenia as a typical social characteristic of Eastern European countries.

The findings indicate that withdrawal significantly impacts women’s sexual satisfaction, particularly about pleasure and orgasm. However, this must be considered because nearly half of the participants used withdrawal out of necessity rather than preference, which may have influenced their experiences and perceptions. Participants reported various effects of withdrawal on their sexual desire and arousal. A decrease in sexual desire was attributed to fear of getting pregnant. However, arousal disorder resulted from insufficient relaxation of a woman who focused on a man’s performance. The orgasmic disorder was the most prevalent disorder among participants, according to the study; withdrawal may disrupt the climax experience in females [8]. Contrary to the findings by Fu et al. [17], participants using withdrawal did not find extra-vaginal ejaculation stimulating or arousing. Although the study investigated the use of withdrawal as a contraceptive method, one respondent highlighted extra-vaginal ejaculation as sex play, which added to greater pleasure. Furthermore, none of the participants considered that withdrawal is better than the use of a condom, nor did any find it more pleasurable, which contradicts assertions in existing literature [18]. Instead, they expressed greater pleasure in sex using more modern and reliable contraceptive methods.

A primary aim of this study was to examine the underlying reasons for the continued use of withdrawal among women, despite its potential negative impact on sexual satisfaction and intercourse quality. The results showed that the reasons originated from traditional contraceptive practices based on social influence. In the past, women often accepted withdrawal at the beginning of their sexual lives without questions, influenced by partners or peers [13]. Prior research indicates that women’s choice of withdrawal may not always have been autonomous but was sometimes adopted to meet partner preferences [13, 19]. Across settings, women reported acquiescing to withdrawal because patriarchal socialisation normalised deference to male sexual decision-making, because they feared relational costs—loss of affection, intimacy, or even abandonment—if they resisted, and because some framed compliance as an altruistic gesture that enhanced their partner’s sexual pleasure. These motives mirror recent evidence that male partners’ power can override women’s contraceptive preferences in Zambia [20], that U.S. women often use non-preferred methods when relational power is unequal [21], and that shifts in partner decision-making power are critical to improving contraceptive agency in Uganda [22]. Many participants reported not consulting gynaecologists before their first sexual intercourse, often due to lack of knowledge, legal age restrictions, fear of societal judgment or difficulty getting medical advice. Historically, gynaecologists occasionally recommended withdrawal, particularly during lactation, based on the belief that other contraceptive methods posed potential risks [5]. This reflects earlier medical perspectives and societal preferences for natural contraception. However, contemporary evidence confirms that modern progestogen contraceptive methods are safe for use during lactation [7].

This historical endorsement clashes with a contemporary medical view, which tends to stigmatise withdrawal [8]. Gynaecologists’ support for withdrawal-initiated discussions about its use and provided women with appropriate information about efficacy and limitations [5]. Other reasons for using withdrawal revealed in the study included missed birth control pills and medical conditions. A subset of participants framed their reliance on withdrawal within a broader “natural-living” ethos, deliberately avoiding hormone-based contraceptives that they perceived as carrying immediate and future health risks—a pattern that recent qualitative and mixed-methods studies in Western settings and Iran likewise attribute to the growing cultural valorisation of naturalness in reproductive health decision-making [23]. This trend is characterised by an idealisation of nature as inherently beneficial, often overshadowing the potential risks of natural processes and practices. This observation aligns with the prevalent social belief in Slovenia and globally that advocates for a “naturalistic” approach across various life domains, i.e., naturalism. Often depicted as contemporary, this belief ostensibly validates and rationalises traditional paradigms. Such a phenomenon contributes to the re-traditionalization of sexual norms and the roles of women in private and societal contexts [24, 25]. It is presumed that the promotion of natural methods like withdrawal serves to perpetuate traditional views under the guise of modernity, thereby reinforcing historical gender roles and sexual dynamics.

Given the fact that withdrawal is a prevalent contraceptive method, it is crucial to elucidate the reasons underlying its selection. A comprehensive understanding of these factors can enhance the effectiveness of contraceptive counselling targeted at women who eschew hormonal and chemically based contraceptive options. Our findings suggest that, among women who already experience sexual dysfunction—particularly orgasmic difficulties—the use of withdrawal may exacerbate these problems, rather than being inherently unsuitable for all women. Furthermore, an informed discourse regarding withdrawal extends beyond the mere consideration of pregnancy risk. It prompts a broader examination of additional concerns that women might not spontaneously express. This underscores the importance of a nuanced approach in contraceptive counselling, wherein the implications of withdrawal on sexual health and satisfaction are thoroughly addressed.

The study’s methodology limits its generalizability as it exclusively involves female participants, thereby overlooking male perspectives on withdrawal. The scope of findings relates solely to women, omitting insights into potential benefits for men despite withdrawal’s reliance on male ejaculation control. The predominance of counselling targeting women, often in gynaecological settings, further neglects male educational needs and viewpoints regarding withdrawal. This limitation underscores the need for inclusive research incorporating male perspectives to provide a more comprehensive understanding of withdrawal’s motivations and impacts across genders. Future studies should engage both male and female participants to assess withdrawal’s role and acceptance in sexual health and relational dynamics.

To sum up, withdrawal has a significant impact on women’s sexual satisfaction, particularly pleasure and orgasm, due to the presence of far for pregnancy and arousal disorder. Although the use of withdrawal in the past was encouraged by sociality and limited birth control options, many women nowadays practice withdrawal to follow the trend towards naturalism, despite its limited effectiveness and potential risks. In the future, comprehensive contraceptive counselling should consider personal and cultural factors which influence women’s decisions. A core component of comprehensive sexuality education is the inclusion of instruction on contraceptive methods, sexually transmitted infection (STI) risk, and partner communication, all of which empower individuals to make informed sexual-health decisions.

## Data Availability

The datasets generated and analysed during the current study are available from the corresponding author on reasonable request.
